# Clinical, Electrodiagnostic Findings and Quality of Life of Dogs and Cats with Brachial Plexus Injury

**DOI:** 10.3390/vetsci7030101

**Published:** 2020-07-31

**Authors:** Marika Menchetti, Gualtiero Gandini, Beatrice Bravaccini, Maurizio Dondi, Teresa Gagliardo, Ezio Bianchi

**Affiliations:** 1 Department of Veterinary Medical Sciences, University of Bologna, 40064 Ozzano dell’Emilia (BO), Italy; gualtiero.gandini@unibo.it (G.G.); beatricebravaccini@libero.it (B.B.); teresagagliardo@hotmail.it (T.G.);; 2 San Marco Veterinary Clinic, Neurology and Neurosurgery Division, 35030 Veggiano (PD), Italy; 3 Department of Veterinary Medical Science, University of Parma, 43121 Parma (PR), Italy; maurizio.dondi@unipr.it (M.D.); ezio.bianchi@unipr.it (E.B.)

**Keywords:** brachial plexus injury, brachial plexus injury, dog, cat, neuropathic pain

## Abstract

Brachial plexus injury (BPI) represents a common consequence of road traffic accidents in humans and small animals. In humans, neuropathic pain is a common symptom after BPI. The aim of the study was to describe the clinical signs, the electrodiagnostic findings, the outcome and the quality of life (QoL) of a cohort of dogs and cats with BPI. Clinical records of 40 dogs and 26 cats with BPI were retrospectively reviewed. Specific attention was put on the evaluation of electrodiagnostic findings (35/40 dogs; 14/26 cats) and telephonic interview results (26/40 dogs; 18/26 cats). The most common neurological condition was the inability to bear weight and sensory deficits on the affected limb. Radial and ulnar motor nerve conduction studies (MNCSs) were absent respectively in 47% (radial) and 62% (ulnar) of dogs and 57% (radial) and 57% (ulnar) of cats. The absence of radial (*p* = 0.003) and ulnar (*p* = 0.007) MNCSs in dogs and ulnar MNCSs in cats (*p* = 0.02) was significantly associated to the amputation of the affected limb. The owners described signs of pain/discomfort in 73% of dogs and 56% of cats. This is the first report suggesting that neuropathic pain/discomfort should be adequately considered in order to improve the QoL.

## 1. Introduction

The results of this study were presented at the 31^st^ ESVN-ECVN symposium, Copenhagen, Denmark, September 20–22, 2018 (Menchetti, M.; Gandini, G.; Dondi, M.; Bravaccini, B.; Gagliardo, G.; Galli, G.; Bianchi, E. Clinical, electrodiagnostic findings and quality of life of dogs and cats with brachial plexus injury. Proceedings of 31st ESVN-ECVN symposium, Copenhagen, Denmark, 20-22; *J Vet Int Med* **2019** Dec 21.doi:10.1111/jvim.15647).

Brachial plexus injury (BPI) represents a quite common clinical entity in humans and small animals, usually as the result of road traffic accidents [1,2,3,4,5,6,7]. BPI is the consequence of severe abduction and/or traction of the thoracic limb and can be partial or complete. Usually, the nerve roots are more easily damaged than the peripheral nerves. This is probably due do their lack of perineurium and the subsequent lower capacity to resist stretch [4,5,6].

BPI always carries a guarded to poor prognosis for functional recovery, which may be different according to the severity of the nerve roots avulsion and, in some cases, require the amputation of the affected limb [3,4]. Prognostic indicators of poor functional recovery of the affected limb in dogs are the lack of pain perception, unilateral loss of the cutaneous trunci reflex and abnormal motor nerve conduction studies (MNCS) of the radial nerve [4,5]. For this reason, electromyography (EMG) and nerve conduction studies can be considered helpful prognostic techniques in dogs and cats affected by BPI [5,6].

In human medicine, pain is a common symptom after BPI, affecting up to 70% of patients. In most cases, pain is predominantly neuropathic and has the greatest negative impact on the quality of life (QoL) of affected patients [7,8]. To date, no data are available in veterinary literature regarding QoL in small animals with BPI. Hence, the primary aims of this study were to describe the clinical signs, electrodiagnostic findings and the outcome of a cohort of dogs and cats with BPI. A further goal was the investigation of their quality of life and the possible presence of persistent signs of discomfort considered from the owners’ perspective.

## 2. Materials and Methods

### 2.1. Animal Population

Dogs and cats presented at the Veterinary Teaching Hospital (VTH) of the Department of Veterinary Medical Sciences of the University of Bologna and at the VTH of the Department of Veterinary Medical Sciences of the University of Parma between January 2004 and December 2017 with a history or suspicion of trauma and clinical and neurologic signs of BPI were retrospectively included in the study. Dogs and cats without complete medical records and thorough neurological examination were excluded from the study.

Data regarding age, breed, sex, bodyweight, affected limb, physiotherapy, eventual amputation of the affected limb, neurologic signs, time elapsed between trauma and neurologic evaluation and, when available, electrodiagnostic findings and time elapsed between trauma and electrodiagnostic evaluation were collected.

All the information and clinical data regarding the animal population included in this study were used according to the Informed Consent the owners signed at the moment of the clinical evaluation.

### 2.2. Neurologic Examination

For each animal, a complete neurologic examination was carried out at the time of admission by a board-certified neurologist (GG, EB) or a resident (MM). Specific attention was put on the evaluation of the severity of the neurological condition. For this purpose, a score from 0 to 4, as previously described [4] was used at the time of admission after the neurological examination: grade 0: normal use of the affected limb; grade 1: weight-bearing monoparesis; grade 2: non weight-bearing monoparesis, maintaining possible flexion of the elbow and shoulder; grade 3: no weight-bearing monoparesis/plegia, pain perception present and grade 4: no weight-bearing monoparesis/plegia, absent pain perception.

Furthermore, the presence of the following neurologic findings was recorded: proprioceptive deficits on the affected limb, Horner’s syndrome, cutaneous trunci reflex, pain perception of the affected limb, licking of the affected limb and self-mutilation.

Cranial (C6-C7), caudal (C8-T2) or complete (C6-T2) brachial plexus injury was diagnosed based on specific neurologic signs, including the ability to move the affected limb, the presence or absence of sensation of the different autonomous zones of the limb, Horner’s syndrome, cutaneous trunci reflex and, when available, electrodiagnostic findings.

### 2.3. Electrodiagnostic Tests

Electrodiagnostic evaluation of the affected limb was performed under general anesthesia using EMG equipment (Myoquick, Micromed, Treviso, Italy). Electromyography was performed recording spontaneous activity from the supraspinatus, infraspinatus, biceps brachii, triceps brachii, carpal extensor, carpal flexor and interosseous muscles of the affected limb with a concentric needle electrode. The type and distribution of the spontaneous EMG activity were retrospectively evaluated for each animal included in the study.

Motor nerve conduction studies (MNCS) were performed on the affected limb and consisted of an orthodromic stimulation of the radial and ulnar nerve and recording of the resultant compound muscle action potentials (CMAPs), as previously described [9]. Sensory nerve conduction studies (SNCS) of the same nerves of the affected limb were performed and retrospectively evaluated. F-waves, magnetic motor evoked potentials (MMEP) and cord dorsum potentials (CDP) were retrospectively evaluated, when available.

### 2.4. Questionnaire

A survey was designed. Particular attention was put on the evaluation of specific signs possibly associated with the presence of pain, the functional outcome, the owner’s perspective of the quality of life of their animals and the impact of BPI on the patient and owner’s daily activities. The survey was submitted to those owners who gave their informed consent. For each case, data were collected by a telephonic interview at least 6-month after the occurrence of BPI.

Regarding the information obtained during the telephonic questionnaire, the owners were asked on the phone to give their consent to use the information for a research study. If they did not agree then the questionnaire was not used for writing this manuscript.

### 2.5. Clinical Outcome

Information regarding follow-up, physiotherapy performed and amputation of the affected limb were retrospectively reviewed. For the purpose of the study, the outcome was considered “poor” if the animal showed no motor function recover at all or if the affected limb was amputated as a result of the brachial plexus injury.

### 2.6. Statistical Analysis

A descriptive statistic was performed for breed, age, gender, weight, site of the lesion, cause of trauma, time elapsed between trauma and neurologic examination, time elapsed between trauma and electrodiagnostic evaluation, neurologic findings, electrodiagnostic evaluation, type of BPI, physiotherapy and amputation of the affected limb. The associations between categorical variables were assessed using the chi-squared test or Fisher’s exact test depending on whether the value in one or more of the cells of the contingency table was five or less. The normality distribution of the data was evaluated using the Shapiro–Wilk test. In the case of normal data distribution, mean and standard deviation were considered. In the case of non-normal distribution, data were reported using the median and range of distribution. Odds ratios (OR) and 95% confidence intervals (CIs) were calculated for variables that did not contain 0 as value. *p* values were considered significant when < 0.05 and when the 95% CI of the OR excluded 1.0. Data analysis was performed using statistical analysis software (PAST 3.x The past of the future, Hammer and Harper, Natural History Museum, University of Oslo, Oslo, Norway), while calculations and graphs were obtained using an electronic spreadsheet (Microsoft Excel, Microsoft Corporation, Microsoft Redmond campus, Redmond, Washington, United States).

## 3. Results

### 3.1. Study Population

Forty dogs and 26 cats were included in the study. Dogs had a median age of 1.9 years (range 0.16–13.7 years). Twenty-one dogs (21/40; 53%) were males (8/21 (38%) neutered) whereas 19/40 (48%) were females (11/19 [58%] spayed). Twenty dogs (20/40; 50%) were pure breed dogs comprising of English Setter (*n* = 2), Cane Corso dog (*n* = 2), German Shepherd (*n* = 2), Jack Russell terriers (*n* = 2) and Setter Gordon (*n* = 2) and one of each of the following breeds: Labrador retriever, Cocker Spaniel, Dachsbracke, Border Collie, Doberman Pinscher, Pitbull, Pinscher, Segugio Italiano, Maremma sheepdog and Caucasian shepherd dog. The median weight of the dogs included was 16 kg (range: 5–50 kg). Twenty-one dogs (21/40; 53%) had a left-sided lesion and 19 dogs had a right-sided lesion (19/40; 47%). In the majority of dogs, the main cause of the BPI was a traffic road accident (31/40; 78%), including a further 5% (2/40) in which this cause was suspected. In the remaining seven dogs, the BPI was caused by falling from above (3/40; 7%), wild boar bite (1/40; 3%) and, in three dogs (3/40; 7%), the cause of the trauma remained unknown.

Cats had a median age of 0.8 years (range 0.1–11 years). Sixteen cats (16/26; 62%) were males (7/16, 44% neutered) whereas 10/26 (38%) were females (5/10, 50% spayed). All cats included were European Shorthair cats. The median weight of the cats was 3.1 kg (range: 0.8–8 kg). 14 cats (14/26; 54%) had a right-sided lesion and 12 cats (12/26; 46%) had a left-sided lesion. In twelve cats (12/26; 46%) the BPI was caused by a traffic road accident, two cats (2/26; 8%) had a limb entrapment and in the remaining twelve cats (12/26; 46%) the original cause of the trauma remained unknown.

### 3.2. Neurologic Examination

The median time elapsed from the trauma to the neurologic examination was 30 days (range 5–157 days) for dogs and 7 days (1–540 days) for cats. The majority of dogs (26/40; 65%) and cats (14/26; 54%) were presented with the most severe neurologic condition with the inability to support weight and absence of pain perception (grade 4; Table 1). Regarding the localization of the BPI, it was considered complete in 23 dogs (58%) and 15 cats (58%), caudal in 16 dogs (40%) and 11 cats (42%) and cranial in 1 dog (2%). None of the cats had a cranial BPI. Partial Horner’s syndrome was described in 24 dogs (60%) and 12 cats (46%) and the absence of ipsilateral cutaneous trunci reflex was reported in 29 dogs (72%) and 15 cats (58%). In 26 dogs (65%) and 19 cats (73%) the absence of pain perception of the affected limb was described. Ten dogs (25%) and 3 cats (12%) showed signs of paresthesia (licking and/or chewing) of the affected limb at the time of the neurologic examination. Furthermore, three dogs (7%) and one cat (4%) showed self-mutilation of the affected limb at the time of neurologic examination (Table 2).

### 3.3. Electrodiagnostic Evaluation

The median time elapsed from the trauma to the electrodiagnostic evaluation was 39 days (range 5–250 days) for dogs and 17 days (4–540 days) for cats. The electrodiagnostic evaluation was performed in 35 dogs (87%) and 14 cats (54%).

The EMG was performed in all 35 dogs and 14 cats and demonstrated spontaneous muscular activity, comprising of fibrillation potentials and positive sharp waves, in the affected limb of each animal.

The radial and ulnar MNCS were evaluated in 34/35 (97%) dogs and all 14 cats. The CMAPs of radial MNCS were not recordable in 16 dogs (47%) and 8 cats (57%), reduced in amplitude in 16 dogs (47%) and 6 cats (43%) and normal in 2 dogs (6%). The CMAPs of ulnar MNCS were absent in 21 dogs (62%) and 8 cats (57%), reduced in amplitude in 12 dogs (35%) and 5 cats (36%) and normal in 1 dog (3%) and 1 cat (7%) (Table 3).

Sensory nerve conduction study (SNCS) was evaluated in 18/35 dogs (51%) and 8/14 cats (57%). The SNCS was normal in 2 dogs (11%) and 5 cats (62%), recordable only for the radial nerve in 3 dogs (17%), recordable only for the ulnar nerve in 8 dogs (44%) and one cat (13%) and not recordable in 5 dogs (28%) and 2 cats (25%) (Table 3).

F-waves test was performed in 11 dogs (31%) and one cat (7%). Of those, F-waves were recordable in the cat and 7 dogs (64%). Cord dorsum potentials (CDPs) were registered in 7 dogs (20%). Of those, CDPs were considered normal in 5 dogs (71%). The CDP was not registered in cats (Table 3).

Magnetic motor evoked potentials (MMEP) were evaluated in 7/35 dogs (21%) and 1/14 cat (7%). MMEPs showed reduced amplitudes in 6 dogs (86%) and were absent in 1 dog (14%) and in the cat (Table 3).

### 3.4. Questionnaire

The median time elapsed from the trauma to the telephonic interview was 5.4 years (range 0.8–11 years) for dogs and 4.7 years (range 0.5–8 years) for cats. Owners of 26 dogs (65%) and 18 cats (69%) answered the questionnaire. Of those, 16 dogs (62%) and 12 cats (67%) were still alive at the time of the questionnaire. Of those that were not still alive (dogs 10/26, 38%; cats 6/18, 33%), 8 dogs (80%) and all 6 cats were euthanized due to other conditions not related to the trauma or BPI. Of the remaining 2 dogs, 1 (10%) was euthanized because of the BPI and 1 (10%) was euthanized for the severity of the general condition related to the trauma.

Data regarding physiotherapy were available in 34 dogs (34/40, 85%) and 19 cats (19/26, 73%). However, precise information, as time intercourse between trauma and physiotherapy or if there was any improvement, was available only for those answering the questionnaire. Hence, only those data were considered for this study. Seventeen dogs (65%) and six cats (33%) underwent physiotherapy, with a median time elapsed from the trauma to the physiotherapy of 14 days for dogs (range 7–60 days) and cats (range 14–30 days). The median duration was 56 days for dogs (range 2-75 days) and 60 days for cats (range 2–90 days). The majority of dogs (13/17, 76%) and cats (5/6, 83%) had a day-hospital regimen during the physiotherapy protocol. According to the owners, physiotherapy facilitated the functional improvement of the affected limb in a total of 7 dogs (41%) and 5 cats (83%). Five cats (83%) that underwent physiotherapy had a neurologic grade from 0 to 3, whereas the majority of dogs that underwent physiotherapy (12/17, 71%) had a neurologic grade of 4.

Nineteen dogs’ (73%) and 10 cats’ owners (56%) noticed behavioral manifestations possibly associated with the presence of pain/discomfort after the BPI. In particular, owners described animals licking (dogs 15/19, 79%; cats 6/10, 60%), looking at (dogs 13/19, 68%; cats 5/10, 50%) and chewing the affected limb (dogs 6/19, 32%; cats 1/10, 10%). Cats showed increased restlessness (4/10, 40%), anxiety (3/10, 30%) and frequent vocalization (4/10, 40%). Interactions with people and other animals were also impaired in terms of aggressive behavior toward people (dogs 2/19, 10%) and toward other animals (dogs 3/19, 16%). Daily activities were compromised in 16 dogs (62%) and 10 cats (56%) in terms of activity level (dogs 15/16; cats 7/10) interactions with animals or people (dogs 4/16; cats 5/10), playfulness (dogs 1/16; cats 1/10) and sleep behavior in cats (1/10).

The overall quality of life was considered excellent in 8 dogs (34%) and 12 cats (67%). Only in 4 dogs (16%) and 1 cat (5%) owners described the QoL of their pets as poor.

After the BPI, some owners of dogs and cats with BPI expressed a feeling of limited independence (dog owners 4/26, 17%; cat owners 3/18, 18%), felt that their pet caused conflicts in their work or daily activities (dog owners 7/26, 29%; cat owners 3/18, 18%) and felt that their social life was limited (dog owners 6/26, 25%; cat owners 3/18, 18%). After the trauma, 54% of dogs’ owners (13/26) and 47% of cats’ owners (8/18) reported an improvement in the quality of their relationship with their pets.

### 3.5. Outcome and Factors Related to the Amputation of the Affected Limb

**Figure 1:**
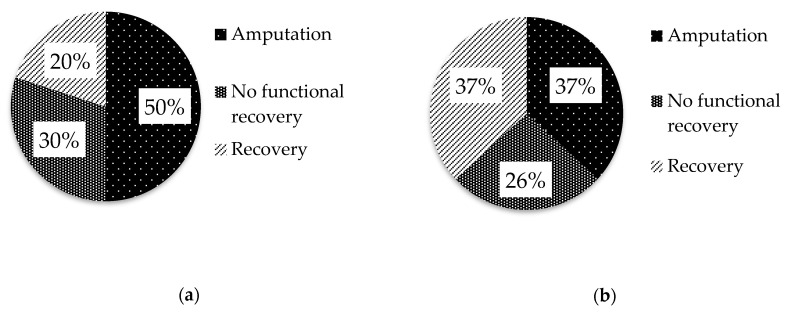
Representation of dogs (**a**) and cats (**b**) with brachial plexus injury; (**a**) 15/30 (50%) of dogs underwent amputation of the affected limb, 30% (9/30) showed no functional recovery of the affected limb and 6/30 (20%) recovered the motor function of the affected limb and (**b**) 7/19 (37%) of cats underwent amputation of the affected limb, 26% (5/19) showed no functional recovery of the affected limb and 7/19 (37%) recovered the motor function of the affected limb.

Medical data of 30 dogs (75%) and 19 cats (73%) were available regarding the outcome. Of these, 24 dogs (80%) and 12 cats (63%) had a poor outcome, as they underwent amputation of the affected limb because of the absence of functional recovery (dogs 15/30, 50%; cats 7/19, 37%) or did not recover the motor function (dogs 9/30, 30%; cats 5/19, 26%; Figure 1).

Among the population of non-amputated dogs (15/30, 50%), 6/15 (40%) recovered the motor function within 3 months after the BPI either completely (2/6, 13%) or partially (4/6, 27%). The level of recovery (partial or complete) remained unchanged throughout the following 12 months after the BPI (Table 4). In the group of non-amputated cats (12/19, 63%), 3/12 (25%) recovered the motor function within 3 months after the BPI (complete 1/3; partial 2/3), 6/12 (50%) recovered the motor function within 6 months after the BPI (complete 3/6; partial 3/6) and 7/12 (58%) recovered the motor function within 12 months after the BPI (complete 4/7; partial 3/7).

In dogs and cats, nor the physiotherapy neither the neurologic grade were statistically related to the possibility of functional recovery (*p* > 0.05). However, the majority of dogs (7/9, 78%) and cats (4/5, 80%) that did not recover were presented with grade 4. No significant relationship between other types of neurologic findings and recovery of the motor function of the limb was seen in dogs and cats.

Despite 87% of dogs (13/15) and 71% of cats (5/7) in which the amputation was performed had a grade 4 lesion (inability to bear weight and absence of pain perception), the neurologic grade at the time of presentation was not statistically related to the amputation of the affected limb (dogs *p* = 0.2; cats *p* = 0.8).

Dogs with the absence of pain perception were 9 times more likely to be amputated than other dogs with intact pain perception (*p* = 0.02). Despite there was no relation with the amputation, all dogs that showed signs of paresthesia, like licking and/or chewing the affected limb, had a severe neurologic condition (grade 4; *p* = 0.02).

No significant relation between other types of neurological clinical findings and amputation of the limb was observed.

Absence of radial (*p* = 0.003) and ulnar (*p* = 0.007) MNCS in dogs and the absence of ulnar MNCS in cats (*p* = 0.03) were significantly related to the amputation of the affected limb. No significant relation between other types of electrodiagnostical findings and amputation of the limb was observed.

The presence of signs of pain/discomfort, the impairment of daily activities, or the QoL of dogs and cats were not statistically related to the amputation of the affected limb.

## 4. Discussion

This study provided a detailed description of a cohort of dogs and cats with traumatic BPI, including the long-term follow-up. Besides the confirmation of findings documented in the veterinary literature, our study reported some new information for this well-known neurologic condition, including the absence of radial and ulnar MNCS in cats as a negative prognostic indicator for the amputation of the affected limb, the description of previously unreported signs after injury that may be interpreted as an expression of residual pain and/or discomfort and the improvement continuing after several months from the injury in a relevant percentage of cats.

The avulsion of the nerve roots represents the most common lesion involving the brachial plexus, usually associated with traumatic injuries, such as road traffic accidents or falling from above, causing severe abduction and/or caudal traction of the thoracic limb [5,10]. In our study, the most common cause of BPI in both dogs and cats was represented by road traffic accidents, confirming what is described in the veterinary literature [1,5,11].

The diagnosis of BPI is usually made on the basis of the characteristic neurologic signs at the time of admission. Prognosis depends on the severity of the clinical presentation, reflecting the grade of nerve roots injury, and, in worst cases, can lead to the lack of functional recovery and amputation of the affected limb [3,4,5]. In our study, in line with what was previously described [4], the absence of pain perception was the single neurologic sign associated to the amputation of the affected limb.

Electrodiagnostic evaluation is considered a useful tool for the assessment of the severity of the nerve root injury [4,12]. Our study confirmed the absence of recordable radial and ulnar MNCS in dogs as a negative prognostic factor related to the amputation of the affected limb [5]. Interestingly, our results documented also the absence of ulnar MNCS significantly related to the amputation of the affected limb in cats after BPI. The superiority of ulnar MNCS compared with radial MNCS as a predictor for functional recovery of the limb was partially unexpected. Possible reasons include the low number of cats included in the study and the different functional importance of the ulnar nerve in cats compared to dogs.

After a traumatic injury, the time needed to develop signs of denervation is 4–5 days, whereas nerve conduction studies are within normal limits for the first few days after trauma [13]. In the present study, the possibility to lack detecting electrodiagnostic abnormalities was quite low because the electrodiagnostic evaluation was performed after a median of 39 days for dogs and 17 days for cats.

Physiotherapy plays an important role in the rehabilitation process to enhance the chances of recovery avoiding contractures and maintaining the limb mobility [13,14,15,16,17,18]. To the author’s knowledge, there are no studies addressing the long-term outcome of dogs and cats with traumatic BPI undergoing physiotherapy. In our study, the majority of dogs and a lower number of cats underwent physiotherapy. More than half of the dogs’ owners reported no improvement, while the cat owners noticed, in more than half patients, some improvement of the motor function. This data could be explained considering that physiotherapy was performed mainly on grade 4 dogs, whereas cats had a lesser degree of neurologic severity (grades 0–3). Our data may suggest that physiotherapy may be useful for those patients with partially maintained limb mobility and/or pain perception. Unfortunately, the low numbers prevented having a statistically significance.

Our data regarding the percentage of dogs and cats with a poor outcome confirm what was previously described [4]. It is noteworthy that 3 months from the BPI 40% of dogs and 25% of cats showed an improvement of neurologic conditions and cats kept improving. Using the necessary caution due to the low numbers, this finding may suggest that the re-evaluation of the cats’ neurologic condition should be performed also after several months from the trauma. Supportive therapies and physiotherapies in time scenario are considered fundamental [14,15].

In the present study, most dogs and more than half of cats showed previously unreported behavioral changes like licking, watching/observing or chewing the affected limb or changes in emotional states and anxiety, suggesting the presence of some degree of discomfort. The QoL questionnaire to the owners was aimed to investigate the presence of discomfort in animals as a possible sign of neuropathic pain consequent to BPI. In humans with BPI, neuropathic pain is reported from 30% to 80% of patients [8,16,17,18,19]. In these patients, the presence of neuropathic pain has the greatest negative impact on their quality of life and seems to be highly refractory to treatments [7,20,21,22,23,24,25]. Recent in-vivo studies using rat model of BPI showed that >30% of rats develop neuropathic pain with long-lasting mechanical allodynia and cold allodynia [2,23,25]. Unfortunately, no data are available regarding the occurrence of neuropathic pain in small animals with BPI. A recent study, focused on a phantom complex in amputated animals, reported similar signs describing a reduction of activity levels, playfulness and participation in family life [26]. 

Our preliminary results were aimed to contribute to increase the awareness of the possible presence of neuropathic pain in BPI patients and, in case, adopt appropriate therapeutic regimens. Indeed, according to the results of the questionnaire, the presence of possible neuropathic discomfort did not seem to impact on the QoL, which was described as excellent or good in the majority of dogs and cats. Owners reported limited independence and conflicts with everyday activities. However, these limitations did not interfere with their relationship with their pets that, in some cases, was perceived as improved, possibly due to the reinforcement of the bond after having experienced the trauma.

Most of the limitations of the present study are related to its retrospective nature. Furthermore, the median time elapsed from the trauma to the telephonic interview was 5.4 years. This long time lapse could have lead to possible errors in the owner’s evaluation, with particular regard to noticing some possible behavioral changes. Other limitations include the relatively low number of the enrolled dogs and cats, and the lack of repeated neurologic exams at standardized time-points in order to objectively evaluate the presence/lack of neurologic improvement. The owners’ perception of their animals’ QoL is subjective and may have influenced the results of the questionnaire. Since there are no validated and objective pain scales and surveys for neuropathic pain in dogs and cats with BPI, the authors used a previously published questionnaire on similar items [26].

## 5. Conclusions

BPI is a well-known neurological disorder and prognosis depends on the severity of the injuries to the affected nerve roots. The absence of pain perception on the affected limb is the single neurologic sign associated to a worst scenario, specifically the amputation of the affected limb. The electrodiagnostic evaluation confirms its importance as a diagnostic and prognostic tool, being the absence of radial and ulnar MNCS in dogs and ulnar MNCs in cats positively associated to the amputation of the affected limb. Our study describes previously unreported long-term findings, including signs of improvement observed in cats after six months from the injury. A percentage of BPI patients can produce behavioral signs possibly related with pain and/or discomfort. The ability to recognize behavioral signs potentially indicating the presence of neuropathic pain/discomfort is a necessary step to plan adequate strategies to improve the QoL of the affected animals.

## Figures and Tables

**Table 1:** Number of dogs and cats per category of neurological deficits.

Grade	Dogs	Cats
grade 0 ^1^	0	0
grade 1 ^2^	4/40 (10%)	0
grade 2 ^3^	9/40 (22%)	9/25 (35%)
grade 3 ^4^	1/40 (3%)	3/26 (11%)
grade 4 ^5^	26/40 (65%)	14/26 (54%)

^1^ Grade 0: normal use of the affected limb; ^2^ grade 1: weight-bearing monoparesis; ^3^ grade 2: non weight-bearing monoparesis, maintaining possible flexion of the elbow and shoulder; ^4^ grade 3: no weight-bearing monoparesis/plegia, pain perception present; ^5^ grade 4: no weight-bearing monoparesis/plegia, absent pain perception.

**Table 2:** Number of dogs and cats per category of neurological findings.

Neurological Findings	Dogs	Cats
Localization		
cranial	23 (58%)	15 (58%)
caudal	16 (40%)	11 (42%)
complete	1 (2%)	0
Partial Horner’s Syndrome	24 (60%)	12 (46%)
Absence of Cutaneous Trunci Reflex	29 (72%)	15 (58%)
Absence of Pain Perception	26 (65%)	19 (73%)
Signs of paresthesia	10 (25%)	3 (12%)
Self mutilation	3 (7%)	1 (4%)

**Table 3:** Number of dogs and cats per category of electrodiagnostical findings.

Electrodiagnostical Findings	Dogs	Cats
Radial CMAP:	evaluated in 34/35 (97%)	evaluated in 14/14 (100%)
not recordable	16 (47%)	8 (57%)
reduced amplitude	16 (47%)	6 (43%)
normal	2 (6%)	0
Ulnar CMAP:	evaluated in 34/35 (97%)	evaluated in 14/14 (100%)
not recordable	21(62%)	8 (57%)
reduced amplitude	12 (35%)	5 (36%)
normal	1 (3%)	1 (7%)
Sensory Nerve Conduction:	evaluated in 18/35 (51%)	evaluated in 8/14 (57%)
normal	2 (11%)	5 (62%)
only radial nerve recordable	3 (17%)	0
only ulnar nerve recordable	8 (44%)	1 (13%)
not recordable	5 (28%)	2 (25%)
F-waves	evaluated in 11/35 (31%)	evaluated in 1/14 (7%)
recordable	7 (64%)	1 (100%)
Cord Dorsum Potentials	evaluated in 7/35 (20%)	not evaluated
normal CDP	5 (71%)	
Magnetic Motor Evoked Potentials	evaluated in 7/35 (21%)	evaluated in 1/14 (7%)
reduced amplitude	6 (86%)	0
absent	1 (14%)	1 (100%)

**Table 4:** Number of dogs and cats that recovered the motor function of the affected limb from 3 to 12 months after the diagnosis of brachial plexus injury (BPI).

Dogs/Cats	Recovery at 3 Months Number (%)	Recovery at 6 Months Number (%)	Recovery at 12 Months Number (%)
Dogs	6/15 (40%)	6/15 (40%)	6/15 (40%)
Cats	3/12 (25%)	6/12 (50%)	7/12 (58%)
